# Baicalein—A Potent Pro-Homeostatic Regulator of Microglia in Retinal Ischemic Injury

**DOI:** 10.3389/fimmu.2022.837497

**Published:** 2022-02-21

**Authors:** Li Pan, Ying Hon Sze, Menglu Yang, Jing Tang, Siming Zhao, Irvin Yi, Chi-Ho To, Chuen Lam, Dong Feng Chen, Kin-Sang Cho, Chi-Wai Do

**Affiliations:** ^1^ School of Optometry, The Hong Kong Polytechnic University, Kowloon, Hong Kong SAR, China; ^2^ Schepens Eye Research Institute of Massachusetts Eye and Ear, Department of Ophthalmology, Harvard Medical School, Boston, MA, United States; ^3^ Department of Ophthalmology, West China Hospital, Sichuan University, Chengdu, China; ^4^ Centre for Eye and Vision Research (CEVR), Hong Kong, Hong Kong SAR, China; ^5^ The Hong Kong Polytechnic University Shenzhen Research Institute, Shenzhen, China

**Keywords:** baicalein, neuroinflammation, microglia, retinal ischemia, proteomics, Th17 cell, IL-1β

## Abstract

Retinal ischemia is a common cause of many retinal diseases, leading to irreversible vision impairment and blindness. Excessive neuroinflammation, including microglial activation and T-cell responses, has been identified as a critical factor associated with neurodegeneration in retinal ischemia. Baicalein is a natural flavonoid reported to have broad anti-inflammatory and neuroprotective bioactivities. Herein, the effects of baicalein on microglia activation *in vitro* and *in vivo* were investigated. We found that baicalein exhibited robust anti-inflammatory effect on cultured human and mouse microglia, as demonstrated by decreased induction of pro-inflammatory cytokines and the phosphorylation of phosphoinositide 3-kinase (PI3K) and nuclear factor kappa B (NFκB). Proteomic analysis further unraveled baicalein’s effect on modulating IL-17 signaling pathways and its upstream regulator IL-1β. Intravitreal administration of baicalein in the mouse model of retinal ischemia/reperfusion (I/R) injury attenuated microglial activation and retinal T-cell infiltration, particularly the T helper 17 cells. Additionally, baicalein was shown to exert neuroprotective effects by significantly reducing the retinal ganglion cell (RGC) loss after I/R injury, leading to an improved retinal function and spatial vision. These results suggest that baicalein, a natural flavonoid, acts as a negative regulator of activated microglia and immune responses both *in vitro* and *in vivo*, effectively alleviating neurodegeneration in retinal I/R injury. This finding indicates that baicalein could be a potential therapeutic agent against currently incurable degenerative retinal diseases.

## Introduction

Ischemia is a pathological condition caused by restricted blood supply to tissues, ultimately leading to neuronal death and functional loss in the central nervous system (CNS) (1, 2). Retinal ischemia, which is often referred to as the “stroke of the retina”, can cause irreversible neuronal loss and visual impairment, as observed in glaucoma, diabetic retinopathy, and retinal or optic nerve trauma (3, 4). Until now, there are limited therapeutic options for patients with retinal ischemia. Crucially, recent studies demonstrate that acute ischemia induces a prolonged phase of neurodegeneration, hence presenting a potential therapeutic window for rescuing resulting vision loss (5).

Microglia are the primary resident immune cells and key players of innate immunity in the CNS, providing immune surveillance and neuronal support (6). They rapidly react to stress by becoming activated (7) and displaying characteristic morphological changes. This is often accompanied by the proliferation, migration, and upregulation of pro-inflammatory cytokines that recruit peripheral immune cells to the CNS (8, 9). However, excessive or prolonged activation of the microglia can result in neurotoxicity (5, 10), eventually inducing neuronal death and progressive neurodegeneration. Accumulating evidence from acute retinal ischemia/reperfusion (I/R) studies demonstrated that chronic microglial activation and neuroinflammation play a key role in driving neurodegeneration (5, 11, 12). Therefore, suppression of prolonged microglial activation or driving inflammatory microglia back to their homeostasis may present a critical strategy for neuroprotective therapies.

Baicalein (5,6,7-trihydroxyflavone; C_15_H_10_O_5_; MW 270.2) is a major flavonoid extracted from the roots of the natural herb *Scutellaria baicalensis* Georgi (SB). SB is widely used in the clinic in Asia to treat various diseases and injuries, such as ischemic stroke and COVID-19 (13–15). Previous studies have shown that baicalein exerts a broad spectrum of bioactivity, including anti-oxidation, anti-apoptosis, and anti-excitotoxicity (16). However, the effect of baicalein on microglial-dependent neuroinflammation has not been established. In the present study, we investigated the effects of baicalein on lipopolysaccharide (LPS)-primed human and mouse microglia and in a mouse model of retinal I/R injury. The data strongly support a potent therapeutic effect of baicalein on retinal I/R injury through limiting microglia and T-cell activation and chronic retinal neuroinflammation.

## Materials and Methods

### Mice

Adult C57BL/6J (B6) mice of both sexes (10–12 weeks old) were purchased from Jackson Laboratory (Bar Habor, Maine, US) (Stock No. 000664, RRID : IMSR_JAX:000664). All animals were handled in accordance with the National Institutes of Health and the Association for Research in Vision and Ophthalmology, and all experimental procedures and the use of animals were approved and monitored by the Animal Care Committee of Schepens Eye Research Institute of Massachusetts Eye and Ear.

### Baicalein Preparation

Baicalein (Cat. No. 70610, Cayman Chemical Company, USA) was dissolved in 20% (2-hydroxypropyl)-beta-cyclodextrin (Cat. No. H107, Sigma-Aldrich, St. Louis, MO, USA) to the desired concentrations and prepared freshly before use. Vehicle control solution 20% (2-hydroxypropyl)-beta-cyclodextrin (βcd) was dissolved in PBS and prepared freshly before use.

### Mouse BV2 Microglial Cell Line and Human Microglial Cell Line HMC3 Culture

Mouse microglia BV2 cell line was purchased from the ATCC (Manassas, Virginia, US) (Cat. No. CRL-2469, RRID : CVCL_5745) and cultured in DMEM medium (Cat. No. 11885084, Thermo Fisher Scientific Waltham, MA, USA) supplemented with 10% fetal bovine serum (FBS; Cat. No. F4135-500ML, Sigma-Aldrich) and 50 ng/ml recombinant mouse colony stimulator factor 1 (rmCSF1) (Cat. No. 315-02, PeproTech, Cranbury, NJ, USA). The medium was changed every 3 days. Cells were seeded into six-well plates and stimulated with 1 μg/ml LPS (Cat. No. L6529-1MG, Sigma) for 6 h followed by vehicle (LPS + Veh) or 10 μM baicalein (LPS + Ba). After 48 h of culturing, the supernatants were collected for cytokine arrays (*N* = 2), and cells were fixed for immunostaining or RNAs were extracted for semiquantitative PCR (qPCR) (*N* = 6).

The human microglial cell line HMC3 was purchased from the ATCC (Cat. No. CRL-3304, RRID : CVCL_II76) and cultured in Eagle’s minimum essential medium (ATCC, Cat. No. 30-2003) with 10% FBS (Cat. No. F2442, Sigma-Aldrich). A total of 2,000 cells per well were seeded in a 48-well plate. Activation of HMC3 cells was induced by 100 ng/ml LPS (Cat. No. L6529, Sigma-Aldrich) for 6 h followed by 5 mM adenosine triphosphate (ATP) (Cat. No. R0441, Thermo Fisher Scientific) for 30 min (17). PBS, vehicle, or baicalein (10 μM) was then added after the cells were stimulated with 100 ng/ml LPS for 6 h plus an additional 30 min of ATP treatment. After 48 h of incubation, RNAs were extracted for qPCR (*N* = 5) and supernatants from HMC3 cell cultures were collected for cytokine array assays (*N* = 4).

### Cytokine Assay

Cytokine arrays were conducted as previously described (18). Both HMC3 and BV2 microglia cells were seeded in six-well plates overnight before the experiments. HMC3 cells were divided into four groups: 1) control, 2) 100 ng/ml LPS + 5 mM ATP, 3) LPS + ATP + Veh, and 4) LPS + ATP + 10 μM Ba. After incubating cells with LPS for 6 h followed by 30 min of ATP stimulation as described previously (17), vehicle or 10 μM baicalein was added. After 48 h of incubation, the supernatants of the culture were collected for cytokine array assay following the manufacturer’s instructions [human cells: Proteome Profiler Human Cytokine Array Kit, R&D Systems, (Minneapolis, US) Cat. No. ARY005B; mouse cells: Proteome Profiler Mouse Cytokine Array Kit, Panel A, R&D Systems, Cat. No. ARY006]. Briefly, the mixture of cell culture supernatant and cytokine detection antibody cocktail was incubated with cytokine array membrane overnight at 4°C. After that, the membranes were incubated with streptavidin–horseradish peroxidase (HRP) for 30 min followed by a Chemi Reagent mix. The signals on the membrane were detected either by X-ray film or iBright CL1500 (Thermo Fisher Scientific). The expression level of cytokines on the membrane was quantified by measuring the pixel density of spots using ImageJ. The value of each dot was normalized to the maximum value for each cytokine. With that, a ratio between 0 and 1 was calculated for each cytokine and used for heatmap generation using GraphPad Prism.

### Real-Time Quantitative Polymerase Chain Reaction

Total RNAs were extracted from mouse retinas or cultured cells using ZYMO Research (Irvine, CA, US) Quick-RNA Microprep Kit (Cat. No. R1051, Zymo Research) following the manufacturer’s instructions. RNA was reverse-transcribed using PrimeScript™ RT Master Mix [Cat. No. RR036A, Takara Bio (Kusatsu, Shiga, Japan)]. A mixture of master mix containing cDNA, 2× Master Mix from KAPA SYBR Fast qPCR kit (Cat. No. 07959397001, Kapa Biosystems, USA), and 10 μM of specific primers was used to detect specific gene expression using the Mastercycler ep realplex real-time PCR system (Eppendorf, Westbury, NY, USA). Relative gene expression level was presented in fold changes after being normalized to the housekeeping gene glyceraldehyde 3-phosphate dehydrogenase (*Gapdh*) using the ΔΔCt method. The results were presented as the relative fold change normalized to the naive retina or cells. The primers’ sequences are listed in **Table 1**.

**Table 1:** Primer sequences used in this study.

Species	Gene name	Primer sequence	
Mouse	*Iba-1*	Forward	TCTGCCGTCCAAACTTGAAGCC
		Reverse	CTCTTCAGCTCTAGGTGGGTCT
	*Ym1*	Forward	TACTCACTTCCACAGGAGCAGG
		Reverse	CTCCAGTGTAGCCATCCTTAGG
	*Nox2*	Forward	TGGCGATCTCAGCAAAAGGTGG
		Reverse	GTACTGTCCCACCTCCATCTTG
	*Ptgs2*	Forward	GCGACATACTCAAGCAGGAGCA
		Reverse	AGTGGTAACCGCTCAGGTGTTG
	*Tlr4*	Forward	AGCTTCTCCAATTTTTCAGAACTTC
		Reverse	TGAGAGGTGGTGTAAGCCATGC
	*Il-1α*	Forward	ACGGCTGAGTTTCAGTGAGACC
		Reverse	CACTCTGGTAGGTGTAAGGTGC
	*Il-1β*	Forward	AAC CTG CTG GTG TGT GAC GTT C
		Reverse	CAG CAC GAG GCT TTT TTG TTG T
	*Ccl2*	Forward	CAA CTC TCA CTG AAG CCA G
		Reverse	TTA ACT GCA TCT GGC TGA G
	*Il-6*	Forward	TACCACTTCACAAGTCGGAGGC
		Reverse	CTGCAAGTGCATCATCGTTGTTC
	*Tnfα*	Forward	TTCTCATTCCTGCTTGTGG
		Reverse	TTGGGAACTTCTCATCCCT
	*Gapdh*	Forward	CATCACTGCCACCCAGAAGACTG
		Reverse	ATGCCAGTGAGCTTCCCGTTCAG
Human	*Il-6*	Forward	AGACAGCCACTCACCTCTTCAG
		Reverse	TTCTGCCAGTGCCTCTTTGCTG
	*Il-1β*	Forward	CCACAGACCTTCCAGGAGAATG
		Reverse	GTGCAGTTCAGTGATCGTACAGG
	*Tnfα*	Forward	CTCTTCTGCCTGCTGCACTTTG
		Reverse	ATGGGCTACAGGCTTGTCACTC
	*Gapdh*	Forward	GTCTCCTCTGACTTCAACAGCG
		Reverse	ACCACCCTGTTGCTGTAGCCAA

### Immunohistochemistry

For immunostaining of cultured cells, BV2 mouse microglia were first seeded onto a Nunc^®^ Lab-Tek™ II Chamber Slide™ System (Cat. No. 154534PK, Thermo Fisher Scientific) and cultured overnight in DMEM (Cat. No. 11885084, Thermo Fisher Scientific) with 10% FBS and 50 ng/ml rmCSF1 (PeproTech, Cat. No. 315-02) at 37°C with 5% CO_2_. After that, the samples were fixed in 4% paraformaldehyde (Sigma) for 10 min followed by permeabilization with 0.05% Triton X-100 in PBS for 10 min, and then blocked with 3% normal donkey serum [Cat. No. 017-000-121, RRID : AB_2337258, Jackson ImmunoResearch Labs (West Grove, PA, US)] for 1 h at room temperature. The cells were then incubated with primary antibodies, rabbit anti-pho-NFκB [1:500, Cell Signaling Technology, (Danvers, MA, US) Cat. No. 3033T, RRID : AB_331284] or rabbit anti-pho-PI3K (1:500, Cell Signaling Technology, Cat. No. 4228S, RRID : AB_659940). After washing three times with washing buffer (0.1% Triton X-100, 0.1% Tween-20 in PBS), they were probed by secondary antibodies including Cy2 donkey anti-rabbit (1:1,000, Jackson ImmunoResearch Labs, Cat. No. 711-225-152, RRID : AB_2340612) and Cy3 donkey anti-rabbit (1:1,000, Jackson ImmunoResearch Labs, Cat. No. 711-165-152, RRID : AB_2307443). The glass slides were mounted with Fluoroshield Mounting Medium with DAPI [Abcam, (Cambridge, UK) Cat. No. ab104139]. Imaging was taken by a Leica SP8 confocal microscope.

For immunostaining of tissue, mice were euthanized by inhalation of carbon dioxide. The eyeballs were collected and fixed in 4% paraformaldehyde overnight at 4°C. Retinas were then dissected out and blocked with Mojito buffer (10% normal goat serum, 3% normal donkey serum, 1% BSA, 0.5% Tween-20, 0.5% Triton X-100, and 0.1% sodium citrate buffer) for 2 h at room temperature. Then, the retinas were incubated with primary antibodies, including mouse anti-BRN3a [1:500, Millipore, (Burlington, MA, US) Cat. No. MAB1585, RRID : AB_94166] and rabbit anti-IBA-1 [1:250, Wako (Minato City, Tokyo, Japan), Cat. No. 019-19741, RRID : AB_839504], at 4°C overnight. After washing the retinas three times in washing buffer (0.1% Triton X-100 in PBS), they were incubated with biotin-conjugated antibodies at 1:250 dilution followed by streptavidin-conjugated Alexa Fluor secondary antibody. Biotin-conjugated antibodies used included goat anti-mouse biotinylated [1:250, Vector Laboratories (CA, US), Cat. No. BA-9200, RRID : AB_2336171]. After rinsing three times in washing buffer (0.3% Triton X-100 in PBS), they were incubated with secondary antibodies including streptavidin, Alexa Fluor 546 conjugate (1:1,000, Invitrogen (Waltham, MA, US), Cat. No. S11225, RRID : AB_2532130), and Cy2 donkey anti-rabbit (1:1,000, Jackson ImmunoResearch, Cat. No. 711-225-152, RRID : AB_2340612) at room temperature for 2 h. After washing three times with washing buffer, the glass slides were mounted with Fluoroshield Mounting Medium with DAPI (Cat. No. ab104139, Abcam). Imaging was taken by the Leica SP8 confocal microscope. For IBA-1 imaging in the whole-mounted retina, only the images of IBA-1^+^ microglia in BRN3a^+^ RGC layer were taken with the depth of around 6–8 μm. ImageJ software (National Institutes of Health, Bethesda, MD, USA) was used to quantify the number of BRN3a^+^ RGCs and IBA-1^+^ microglia/macrophage, as well as the measurement of the process length of microglia and cell body size. All quantification procedures were conducted by two researchers in a masked fashion.

### Western Blot

BV2 cells from different groups were lysed with Pierce™ RIPA lysis and extraction buffer (Cat. No. 89900, Thermo Fisher Scientific) and supplemented with 1× Halt protease and phosphatase inhibitor (Cat. No. 1861261, Thermo Fisher Scientific). The samples were then sonicated on ice by a Q55 Sonicator (Qsonica, NY, USA; four pulses of 22 kHz, 5 s each at 20% power output). The lysates were centrifuged at 17,000×*g* for 5 min. The protein concentration of the lysates was determined using Qubit 4 Fluorometer (Thermo Fisher Scientific). A 30-μg protein was loaded per lane and separated by SDS-PAGE (4%–20% Mini-PROTEAN polyacrylamide gel, Cat. No. 4561096, Bio-Rad). The proteins were electrophoretically transferred to 0.45-μm pore nitrocellulose membranes by Trans-Blot Turbo Transfer System (Bio-Rad). The membranes were blocked with 3% BSA (A7096, Sigma-Aldrich) at room temperature for 1 h and then incubated overnight with the primary antibodies at 4°C, including rabbit anti-phospho-NFκB (1:1,000, Cell Signaling Technology, Cat. No. 3033T, RRID : AB_331284), rabbit anti-phospho-PI3K (1:1,000, Cell Signaling Technology, Cat. No. 4228S, RRID : AB_659940), and mouse anti-β-actin (1:4,000, Thermo Fisher Scientific, Cat. No. MA5-15739, RRID : AB_10979409). After washing with PBS-T buffer (PBS with 0.1% Tween 20) for three times, the blots were incubated with HRP-conjugated 1:2,000 secondary antibody in 3% BSA in PBS-T [Bio-Rad Laboratories (Hercules, CA, US), goat anti-rabbit IgG, Cat. No. 170-6515, RRID : AB_11125142; goat anti-mouse IgG, Cat. No. 172-1011, RRID : AB_11125936] for 1 h at room temperature. Signals were developed with ECL using a Super Signal West Pico kit (Thermo Fisher Scientific) and detected with iBright CL1500 (Thermo Fisher Scientific). The densitometric analysis was performed with ImageJ software using 8-bit grayscale positive chemiluminescent membrane images. All the quantification results were averaged from six independent blots per group and expressed as the mean ratio of the values (target protein/housekeeping protein) ± SEM.

### Mass Spectrometry-Based Proteomics Analysis

Proteins were extracted from cultured BV2 cells as described (19). Briefly, cells were subjected to different treatments, including naive, LPS-treated BV2 for 6 h followed by the addition of vehicle or 10 μM baicalein. After a total of 48 h, cells were washed three times with PBS to remove FBS in the culture medium. Then, the cells were homogenized by 100 μl 5% SDS lysis buffer. Then, samples were centrifuged at 16,000×*g* for 30 min at 4°C. The supernatant was recovered for protein concentration measurement using Pierce™ Rapid Gold BCA Protein Assay (Cat. No. A53225, Thermo Fisher Scientific), according to the manufacturer’s protocol. Protein (50 µg) from each sample was reduced with 20 mM dithiothreitol (Cat. No. D0632, Sigma-Aldrich) and then alkylated with 40 mM iodoacetamide (Cat. No. I1149, Sigma-Aldrich) at room temperature in the dark for 10 min. The SDS lysate was acidified to a final concentration of 1.2% aqueous phosphoric acid. The solution was then diluted with the S-Trap protein binding buffer (90% methanol, 0.1 M TEAB, pH 7.55), at 6:1 (ratio, v/v), with S-Trap Mini Spin Column [ProtiFi(Farmingdale, NY, US), Cat. No. C02-mini-40]. Proteins were enzymatically digested with trypsin, 1:25 (w/w, trypsin:protein), according to the manufacturer’s protocol. The eluted peptides were subsequently dried up at 4°C in a vacuum centrifuge (Labconco, MO, USA), and the pellet was redissolved in 0.1% formic acid. The eluted peptide concentration was measured using colorimetric peptide assay (Cat. No. 23275, Thermo Fisher Scientific). Lastly, the peptides (2 µg) in each sample were subjected to nano-liquid chromatography tandem mass spectrometry (LC-MS/MS).

The hybrid quadrupole time-of-flight TripleTOF^®^ 6600 mass spectrometer (Sciex, MA, USA) was employed for target-free Sequential Window Acquisition of all Theoretical Mass Spectra (SWATH-MS) acquisition. SWATH-MS is a data-independent acquisition (DIA) that allows an in-depth, accurate, and consistent label-free quantification of proteins, particularly intracellular proteins. First, 2 µg tryptic peptides were pooled from all samples to establish protein identification and spectral library using data-dependent acquisition (DDA) (20). Then, 2 µg of peptide from each individual sample was injected into LC-MS/MS equipped with a SilicaTip electrospray emitter (New Objective, FS360-20-10-N-20-C12, 10 μm) handled by nano-flow liquid chromatography (NanoLC 415, Eksigent) for SWATH-MS acquisition. The raw data were collected and protein mapping was processed by the Paragon™ algorithms using ProteinPilot 5.0 (Sciex) for protein identification with a false discovery rate (FDR) ≤1%. Quantitative SWATH data were processed using PeakView 2.2 (Sciex) for spectral extraction followed by normalization and statistical analysis using MarkerView 1.3 (Sciex). The statistically significant differential proteins were evaluated with Welch’s *t*-test at *P <*0.05 with at least 2 quantifiable peptides per protein, represented as the mean of fold change of all biological samples (*N* = 6 per group).

The pathway analysis and upstream regulator prediction were performed by QIAGEN Ingenuity Pathway Analysis (IPA). Differentially expressed proteins (DEPs) between the LPS + Veh and LPS + Ba groups as defined by statistical significance (FDR < 0.01) were selected for gene set overrepresentation analysis by Mammalian Phenotype Ontology (MPO) (http://bioinfo.vanderbilt.edu/webgestalt/) (21) and functional enrichment analysis by Gene Ontology (GO), using the online tool of DAVID analysis (http://david.ncifcrf.gov/) (22).

### Acute Retinal Ischemia/Reperfusion Injury

Retinal ischemia was induced in adult mice unilaterally as previously described (5). Mice were anesthetized with a mixture of 120 mg/kg ketamine and 20 mg/kg xylazine in sterile saline (1:1:4). The pupil was dilated by 1% tropicamide eye drop followed by administrating a drop of topical anesthesia 0.5% proparacaine hydrochloride on the cornea. An easy entry hole for a glass micropipette was made by a 30-gauge needle at the peripheral cornea. The glass micropipette connected to a sterile saline bag by an extension tube was cannulated to the anterior chamber through the entry on the cornea. By elevating the saline bag to 1 m above the mouse eye level, the intraocular pressure (IOP) was raised to 75 mmHg. After 50 min of elevated IOP, the glass micropipette was withdrawn from the anterior chamber. Antibiotic ointment was applied on the cornea, and the anesthetized mice were left to recover on a warm pad. Mice were sacrificed at 1 and 4 weeks following I/R injury for qPCR and immunostaining of IBA-1^+^ microglia and at 4 weeks after I/R injury for immunostaining of BRN3a^+^ RGC and IBA-1^+^ microglia. For the quantification of microglia morphology changes, *N* = 4–5 mice per group; for qPCR measurements of mRNA levels of microglia activation markers and pro-inflammatory cytokines, *N* = 6 mice per group; for identifying T-cell population in retina or LNs using flow cytometry, *N* = 5 mice per group; and for RGC quantification and retinal function tests, *N* = 5–8 mice per group. All normal eyes used in this study were from naive mice.

### Intravitreal Injection

Intravitreal administration of vehicle or baicalein was performed weekly post-I/R injury. The first administration was intravitreally given to mice immediately after the completion of retinal I/R model induction. Adult mice were anesthetized by i.p. injection of ketamine (120 mg/kg)/xylazine (20 mg/kg) mixture. A drop of proparacaine HCl (0.5%; Baush & Lomb Incorporated, Tampa, FL, USA) was applied to numb the eye. A hole was punctured on the limbus using a 30-gauge needle. Two microliters of Veh or 10 μM Ba was injected into the vitreous *via* a glass micropipette. Care was taken to avoid damaging the lens or retina. Antibiotic ointment was applied on the entry site on the limbus before leaving the anesthetized mice to recover on a heating pad.

### Flow Cytometry

T subsets as defined by their cytokine expression in the eye’s draining (superior cervical) lymph nodes (LNs) and retina were measured (10). For retinal cell flow cytometry, anesthetized mice were transcranially perfused with 15 ml saline. The retinas were dissected out and digested by papain. After incubating for 15 min at 37°C, papain digestion was stopped by ovomucoid protease inhibitor. Superior cervical LNs were dissected, and cells were mechanically dissociated using two forceps. Cell aggregates were separated by filtration through a 70-µm nylon cell strainer [Corning (Glendale, AZ, US), 431751]. For analyzing the frequencies of CD4^+^ T cells that expressed IFN-γ (Th1) and IL-17 (Th17), isolated lymphocytes were washed in IsoFlow (Beckman Coulter Inc., Brea, CA, USA) and stained with FITC-conjugated anti-mouse CD4 antibody (IgG2b, clone GK1.5, Biolegend, Cat. No. 100406, San Diego, CA, USA). Thereafter, cells were permeabilized with perm/wash buffer (Invitrogen, Cat. No. 00-8333-56) and stained with PE-labeled anti-mouse IFN-γ antibody (IgG1, clone XMG1.2, Biolegend, Cat. No. 505808, RRID : AB_315402) and PE-labeled anti-mouse IL-17A (IgG1, clone TC11-18H10.1, Biolegend, Cat. No. 506901, RRID : AB_315461) to detect Th1 and Th17 cells, respectively. The antibody-stained cells were analyzed with BD LSR II Flow Cytometer (BD Biosciences), and data were analyzed using FlowJo (FlowJo LLC, Ashland, OR, USA).

### Electroretinography

Mice were dark adapted for at least 8 h before the tests. Adult mice were anesthetized by i.p. injection of ketamine (120 mg/kg)/xylazine (20 mg/kg) mixture using a 25-gauge needle. Both pupils were dilated by topical application of 1% tropicamide followed by a drop of Genteal to keep the cornea moist. The mouse was placed on a heated platform kept at 37°C. The reference and ground electrodes were inserted beneath the skin over the forehead and tail base, respectively. Two gold-ring recording electrodes were gently placed on the center of the corneas with a drop of artificial tear (Genteal, Alcon) (Fort Worth, TX, US) covered. The positive scotopic threshold responses (pSTR) were recorded by averaging 40 responses following flash stimulation intensities at 1.7E−4 (cd s/m^2^). The amplitude of pSTR was measured from the baseline to the peak of the positive deflection. Data were processed by the software included in the electroretinography (ERG) recorder system (Espion Electroretinography System; Diagnosys LLC, Lowell, MA, USA). After the ERG recording, antibiotic ointment and artificial tear were applied to the mice cornea, and mice were left on a heated pad until recovery.

### Optomotor Response

Optomotor response was recorded as previously described (23). Briefly, the mouse was placed on a small platform with stimuli presented on the computer monitors. The luminance of individual stripes was measured using a 371 R Optical Power Meter (Graseby Optronics, Orlando, FL, USA) positioned at the eye level. A constant speed of movement (black and white stripes) with adjustable contrast and the width of stripes on the monitors were presented. The contrast of a given spatial frequency was calculated using the formula (*L*_max_ − *L*_min_)/(*L*_max_ + *L*_min_), where *L*_max_ is the luminance (in cd/m^2^) of the white stripe, and *L*_min_ is the luminance of the black stripe (in cd/m^2^). For visual acuity measurements, spatial frequency was determined at a constant speed of 12 degree/s. For contrast sensitivity measurements, the spatial frequency was set to 0.186 cyc/degree at the same speed. The threshold of visual acuity and contrast sensitivity was determined based on the tracking of movements of mouse head in response to stimuli.

### Statistical Analysis

All statistical analysis was performed using GraphPad Prism for Windows, version 9.0 (GraphPad Software Inc, San Diego, CA, USA). Statistical analysis was conducted by either Student’s *t*-test or one-way ANOVA with Tukey’s multiple comparisons test. Two-way ANOVA with Šídák’s multiple comparisons test was used for comparison of multiple time points. *P <*0.05 was considered statistically significant. Data were expressed as mean ± SEM. *N* refers to the number of mice or the number of biological replicates in cell cultures. Each dot in each figure represented individual replicates.

## Results

### Baicalein Suppresses the Activation of Both Human and Mouse Microglia

**Figure 1:**
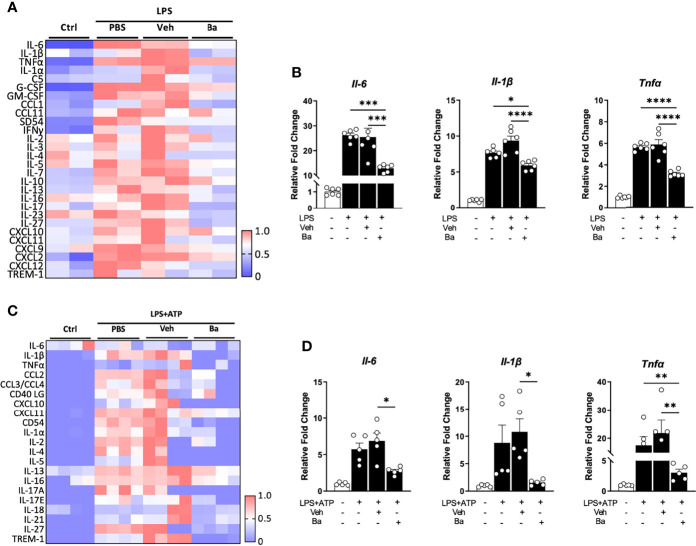
Baicalein suppresses the activation of both human and mouse microglia. **(A)** Heatmap showing the quantification of cytokine expression in the supernatants of BV2 microglial cultures that received no treatment (Ctrl) or treatment with lipopolysaccharide (LPS) followed by PBS, vehicle control (Veh), or baicalein (Ba). The scale bar (0–1) of the heatmaps represents the raw pixel intensities normalized to the maximum intensity for each cytokine. **(B)** qPCR results showing the relative expression of pro-inflammatory cytokines in LPS-stimulated BV2 cells followed by Veh or Ba treatment. *N* = 6/group. Relative fold changes of genes were normalized to naive control group. **(C)** Heatmap showing the quantification results of cytokine protein expression from the supernatants of HMC3 cultures that received no treatment (Ctrl) or treatment with LPS+ATP plus PBS, vehicle (Veh), or baicalein (Ba). **(D)** qPCR results showing the relative expression of pro-inflammatory cytokines in LPS and ATP-stimulated HMC3 cells followed by vehicle (Veh) or baicalein (Ba) treatment. *N* = 5/group. Relative fold change of genes was normalized to naive control group. Data were presented as mean  ±  SEM. Each dot represented individual replicate. *P < 0.05, **P < 0.01, ***P < 0.001, ****P < 0.0001, one-way ANOVA with Tukey’s multiple comparisons test.

As the primary resident immune cells in the CNS, the microglia play a critical role in surveilling and maintaining the homeostasis and functionality of neurons (24, 25). To determine if baicalein has direct effects on microglial homeostasis, we first investigated in LPS-primed mouse BV2 microglia cultures. To comprehensively examine cytokine inductions following LPS and baicalein treatment, we performed pro-inflammatory cytokine screening using cytokine arrays followed by validation at the mRNA levels using qPCR. BV2 cells were incubated with or without LPS (dissolved in PBS), followed by the addition of PBS control, 20% βcd (solvent for dissolving baicalein) as vehicle (Veh) control, or baicalein (Ba) (**Figure 1A**) and cultured for total 48 h. As expected, cytokine arrays of the supernatants detected drastically increased production of all pro-inflammatory cytokines in LPS-treated cultures compared with non-treated controls. Among LPS-primed groups, PBS and vehicle-treated cells showed similar induction of cytokines, suggesting minimal effects of vehicle on LPS-primed BV2 cells. In contrast, the addition of baicalein after LPS stimulation significantly suppressed the production of the most pro-inflammatory cytokines examined, such as IL-6, IL-1β, TNF-α, IL-17, and CXCL10, compared with the vehicle group (**Figure 1A**). The results of qPCR confirmed the induction of mRNAs of the corresponding pro-inflammatory cytokines by LPS and the inhibitory effects of baicalein in BV2 microglia (**Figure 1B**).

The effects of baicalein were also verified in the human microglia HMC3 cell line primed by LPS and ATP (17). After a total of 48 h of incubation, LPS and ATP stimulation induced increased production of pro-inflammatory cytokines, such as IL-1β, IL-17A, IL-1α, CXCL10, and CCL2 as shown by cytokine arrays, compared with the untreated control group (**Figure 1C**). The Veh or PBS-treated group showed a pattern of increased pro-inflammatory cytokine expression similar to that seen in the LPS and ATP-primed group (**Figure 1C**), confirming that vehicle control did not affect microglial activation. Notably, treatment with baicalein brought the majority of inflammatory cytokines nearly to their baseline levels (**Figure 1C**). The qPCR data confirmed that baicalein suppressed the mRNA levels of pro-inflammatory cytokines, such as *Il-6*, *Il-1β*, and *Tnfα*, in LPS and ATP-primed HMC3 cells (**Figure 1D**). These results revealed a direct anti-inflammatory and pro-homeostatic effect of baicalein on microglia.

### Baicalein Inhibits LPS-Induced Phosphorylation of PI3K/NFκB Axis in Microglia

**Figure 2:**
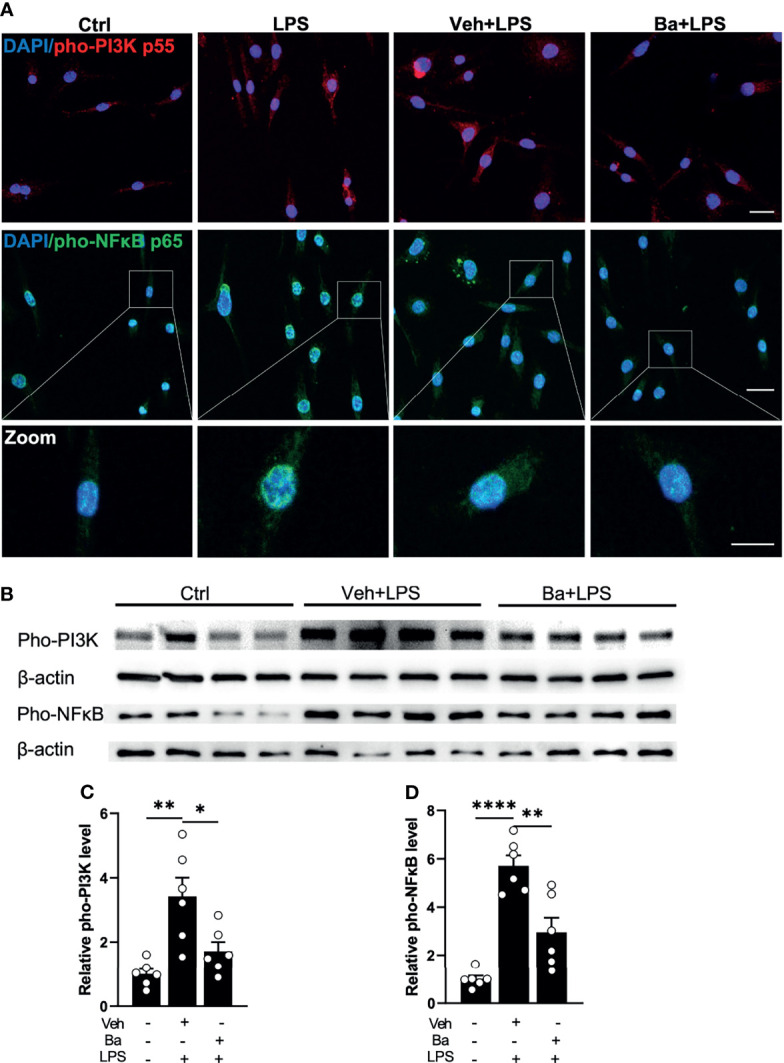
Baicalein inhibits LPS-induced phosphorylation of PI3K/NFκB axis in microglia. **(A)** Representative immunofluorescence photomicrographs showing pho-PI3K p55 (red), pho-NFκB p65 (green), and nuclear labeling with DAPI (blue) on BV2 microglial cells that were untreated (Ctrl) or pretreated with vehicle (Veh) or 10 μM baicalein (Ba) for 24 h followed by 3 h LPS stimulation. Insets showing enlarged images of cells with pho-NFκB and DAPI labeling. Scale bar = 20 μm; 10 μm (insert). **(B)** Western blots showing the expression of pho-PI3K p55 and pho-NFκB p65 and β-actin (loading control) in BV2 cells that were untreated (Ctrl) or pretreated with Veh or Ba for 24 h followed by 3 h LPS stimulation. **(C, D)** Relative densitometer analysis of Western blots for pho-PI3K **(C)** and pho-NFκB **(D)** normalized to β-actin independently in BV2 cells. *N* = 6 per group. Data were presented as mean  ± SEM. Each dot represented individual replicate. *P < 0.05, **P < 0.01, ****P < 0.0001, one-way ANOVA with Tukey’s multiple comparisons test.

Phosphorylation of PI3K and its downstream signal NFκB pathway has been reported to be key intracellular events associated with the pro-inflammatory responses of the microglia (26, 27). To determine if baicalein suppresses the PI3K/NFκB axis, we examined the phosphorylation of PI3K (pho-PI3K) and NFκB (pho-NFκB) in LPS-primed BV2 microglia with immunohistochemistry (**Figure 2A**). Because LPS-induced phosphorylation of PI3K and NFκB (28, 29) is most prominent at 3 h post-LPS stimulation, we pretreated BV2 microglia with vehicle or baicalein followed by LPS stimulation. Increased phosphorylation of PI3K and NFκB induced by LPS was detected in BV2 cells at 3 h post-LPS stimulation, whereas cultures pretreated with baicalein exhibited attenuated pho-PI3K and pho-NFκB levels after LPS priming compared with the LPS and Veh + LPS groups (**Figure 2A**). This result was confirmed by Western blot assays (**Figure 2B**). As expected, Western blot detected significant increases of pho-PI3K and pho-NFκB post-LPS stimulation in vehicle-treated BV2 cells (Veh + LPS) compared with controls (**Figures 2B-D**). In contrast, treatment with baicalein (Ba + LPS) significantly attenuated LPS-induced PI3K and NFκB phosphorylation compared with the Veh + LPS-treated group (**Figures 1B-D**). These results suggest that baicalein is capable of suppressing LPS-induced PI3K and NFκB signaling axis and serving as a pro-homeostatic agent in mouse microglia.

### Proteomic Analysis Further Reveals IL-17 Signaling and Upstream Regulator of IL-1β Inhibited by Baicalein on LPS-Primed Microglia

**Figure 3:**
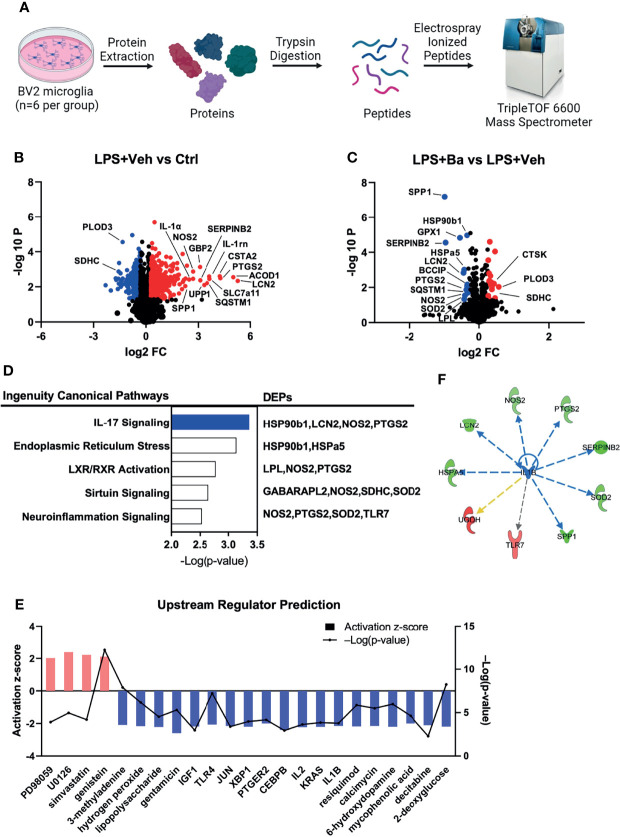
Proteomic analysis further reveals IL-17 signaling and upstream regulator of IL-1β inhibited by baicalein on LPS-primed microglia. **(A)** Graphic illustration of the design of proteomics study. The illustration was created with BioRender. Volcano plot showing the differentially expressed proteins (DEPs) in the groups of **(B)** LPS + Veh vs. Ctrl and **(C)** LPS + Ba vs. LPS + Veh. Red dots represent the upregulated DEPs (*P*-value < 0.05 and FC > 1.2) and blue dots are the downregulated DEPs (*P*-value < 0.05 and FC ≥ 1.2). **(D)** Top 5 canonical pathways (left) enriched by IPA analysis of involved DEPs (right) in BV2 cells (LPS + Ba vs. LPS + Veh). Bar chart was presented with the log-transformed *P*-value, representing the top disease-related biological pathways and involved proteins in each pathway. Blue and white bars represented *z*-score = −2 and 0, respectively. **(E)** Upstream regulator predication by IPA; bar represented the activation *z*-score, and the line chart represented –Log(*P*-value). **(F)** Representative network showed the interaction between the upstream regulator IL-1β and its downstream molecules. Red and green colors represented increased and decreased measurement in the samples. Blue, yellow, and gray lines with arrow represented inhibition, findings inconsistent with state of downstream molecule, and effect not predicted, respectively. *N* = 6 biological samples.

To comprehensively study the intracellular signaling changes and explore the underlying mechanisms of baicalein-mediated microglial responses, we employed the systemic proteomic approach using SWATH-MS acquisition. BV2 cells were treated with LPS followed by the addition of vehicle or baicalein, and they were cultured for a total of 48 h before being collected for proteomic analysis. Equal amounts of reduced peptides from each sample were ionized and electrosprayed into MS for quantification (**Figure 3A**). MS detected 2,445 unique proteins that were mapped to mouse gene IDs with 1% FDR. Among these proteins, we detected 742 DEPs between the LPS + Veh and untreated control cultures, at a cutoff of *P <*0.05 and FC >1.2 (**Figure 3B**). The top 10 upregulated DEPs detected in LPS + Veh cells included LCN2, ACOD1, PTGS2, CSTA2, SLC7a11, IL1rn, SQSTM1, UPP1, SERPINB2, and GBP2. Enriched GO analysis identified biological processes related to oxidation–reduction (e.g., PTGS2, PLOD3, SDHC), lipid metabolic process (e.g., IL-1rn, PTGS2), and immune system process (e.g., ACOD1, LCN2, TLR7) (**Figure 3B** and **Figure S1A**). Enriched MPO analysis showed LPS-induced abnormal phenotypes of adaptive and innate immunity and professional antigen-presenting cell (pAPC) physiology (**Figure S1B**). Among the 742 DEPs induced by LPS stimulation, 28 LPS-induced DEPs (*P* < 0.05 and FC > 1.2) were reversed by the addition of baicalein at 6 h after LPS stimulation (**Figure 3C**). Notably, 4 of the top 10 most significantly upregulated DEPs in the LPS + Veh group, namely, PTGS2, LCN2, SQSTM1, and SERPINB2, were significantly suppressed by baicalein (**Figure 3C**). GO biological processes also linked the baicalein-induced DEPs to oxidation–reduction, lipid metabolic and apoptotic processes, and inflammatory responses, suggesting the reversal of LPS-initiated processes by baicalein (**Figure S1C**). A similar suppression of innate immunity and pAPC was identified in the baicalein-treated group (**Figure S1D**). Canonical pathway analysis of baicalein (LPS + Ba)-induced DEPs performed by IPA further revealed their association with the endoplasmic reticulum (ER) stress and neuroinflammation, specifically through inhibition of the IL-17 pathway (with *z*-score = 2; **Figure 3D**). IL-1β, TLR4, and chemical-kinase inhibitors (e.g., PD98059 and U0126) were predicted as upstream regulators induced by baicalein (**Figures 3E, F** **)**. Significant downregulation of PTGS2, LCN2, SERPINB2, and SPP1 further linked the upstream regulator IL-1β to this regulation process (**Figure 3F**). These results, together with the observed inhibition of IL-1β and IL-17A expression as shown in the above cytokine array and qPCRs (**Figures 1A-D**), strongly support that baicalein inhibits LPS-induced activation of the microglia and IL-17 pathway, *via* IL-1β and TLR4 signaling.

### Baicalein Alleviates Retinal I/R Injury-Induced Retinal Microglia Activation and Neuroinflammation

**Figure 4:**
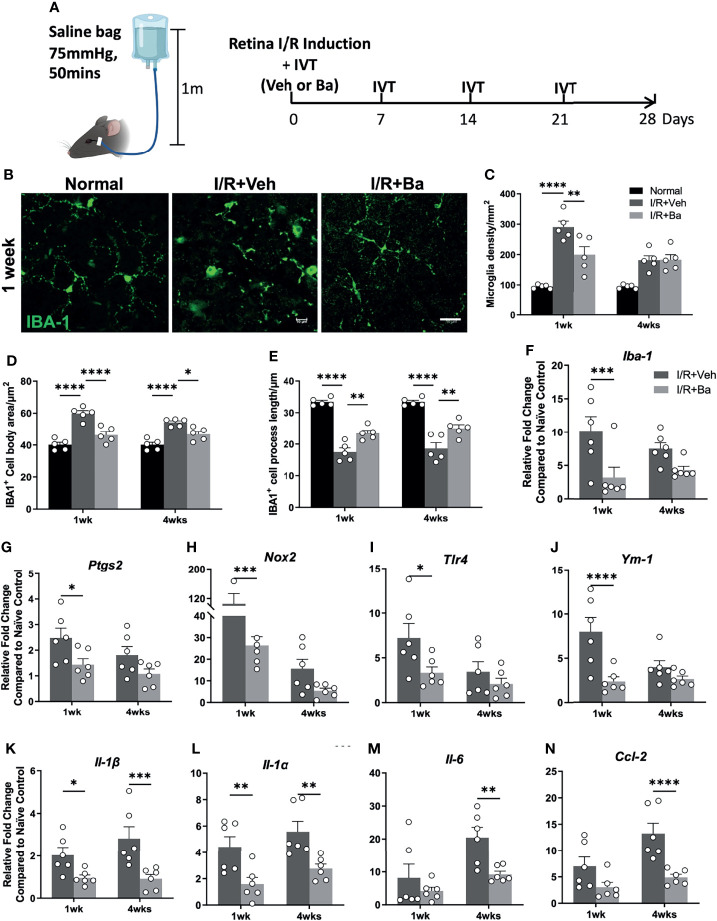
Baicalein alleviates retinal ischemia/reperfusion (I/R) injury-induced retinal microglia activation and neuroinflammation. **(A)** Schematic illustration of the mouse I/R model and the frequency of intravitreal (IVT) injection of vehicle (Veh) or baicalein (Ba). **(B)** Representative immunofluorescence images of IBA-1-labeled microglial cells (green) on flat mount retina from normal, vehicle (Veh), or 100 μM baicalein (Ba)-treated mice at 1 week post-I/R. Scale bar = 20 μm. Quantification of **(C)** microglial density, **(D)** cell body area, and **(E)** process length of Iba-1-positive cells (*N* = 5 per group). qPCR analysis of activated microglia markers **(F–J)** and pro-inflammatory cytokines **(K–N)** in Veh or Ba-treated mice (relative fold changes normalized to naive control) at 1 and 4 weeks post-I/R injury (*N* = 6 per group). Data were presented as mean  ± SEM. Each dot represented individual replicate. *P < 0.05, **P < 0.01, ***P < 0.001, ****P < 0.0001, two-way ANOVA with Šídák’s multiple comparisons test.

To evaluate the anti-inflammatory effect of baicalein in microglia *in vivo*, we induced acute retinal I/R in mice followed by weekly intravitreal administration of vehicle or baicalein (**Figure 4A**). Our previous studies (5, 18) reported that acute retinal I/R leads to neuroinflammation characterized by microglial activation and subsequent infiltration of CD4^+^ T cells into the retina (5, 18). Consistent with our previous findings, we detected robust morphological changes of the microglia on the RGC layer. It featured increased numbers of IBA-1^+^ microglia/macrophages, enlargement of IBA-1^+^ cell body size, and shortened cellular processes at 1 week post-I/R insult (**Figures 4B–E**, and **Figure S2**). At the mRNA level of retina post-I/R injury, we detected increased expression of activated microglial markers (including *Iba-1*, *Ptgs2*, *Nox2*, *Tlr4*, and *Ym1*, **Figures 4F-J**) and pro-inflammatory cytokines (including *Il-1β*, *Il-1α*, *Il-6*, and *Ccl2*, **Figures 4K–N**), compared with the naive group. Remarkably, baicalein treatment retained the morphological features of the resting microglia, as shown by a lower IBA-1^+^ cell density, smaller cell body size, and longer cellular processes in the retina at 1 and 4 weeks post-I/R insults (**Figures 4B–E**, and **Figure S2**). In addition, baicalein significantly suppressed the expression of activated microglial markers including *Iba-1*, *Ptgs2*, *Nox2*, *Tlr4*, and *Ym1* at 1 week after I/R insult, but not at 4 weeks post-injury (**Figures 4F–J**). Significant downregulation of pro-inflammatory cytokines, including *Il-1β*, *Il-1α*, *Il-6*, and *Ccl2*, was also revealed at 1 and 4 weeks post-I/R injuries (**Figures 4K–N**). These results suggest that weekly administration of baicalein effectively suppressed the I/R-induced retinal microglial activation and neuroinflammation.

### Baicalein Attenuates CD4^+^ Effector T Cells Th17 and Th1 Cell Responses in the Retina and Superior Cervical Lymph Nodes After I/R Insults

**Figure 5:**
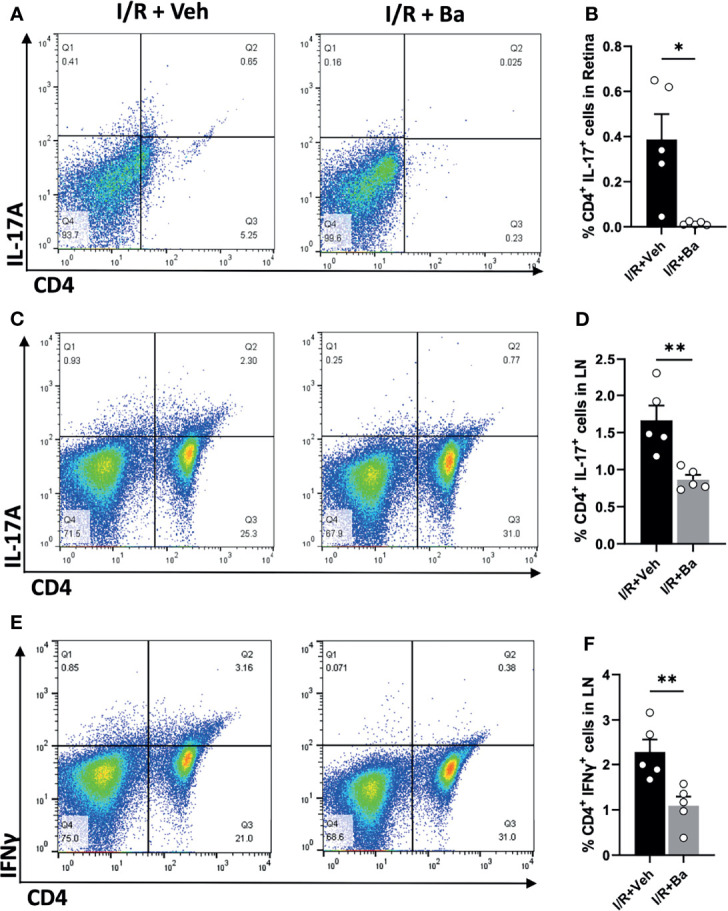
Baicalein attenuates CD4^+^ Th17 and Th1 cell responses in the retina and superior cervical lymph nodes after I/R insults. **(A)** Representative FACS plots showing the gated population of CD4^+^ and IL-17A^+^ T cells and **(B)** their frequency in the retinas from the Veh or 100 μM baicalein (Ba)-treated mice at 2 weeks I/R injury. **(C)** Representative FACS plot of CD4^+^/IL-17A^+^ T cells and **(D)** their frequency in the cervical lymph nodes (LNs) of the Veh or Ba-treated mice at 2 weeks post-I/R injury. **(E)** Representative FACS plots and **(F)** the quantification of the frequency of CD4^+^/IFNγ^+^ T cells in the cervical LNs of the Veh or Ba-treated mice at 2 weeks post-I/R injury. *N* = 5 per group. Data were presented as mean  ± SEM. Each dot represented an individual replicate. *P < 0.05, **P < 0.01, Student’s *t*-test.

Despite the eyes being regarded as an immune-privileged site, it has been shown that the compromise of the blood retinal barrier under stress allows for the recruitment of peripheral immune cells to the lesion site and triggers adaptive immune responses, ultimately aggravating neuronal death (5, 30). We previously reported that retinal infiltration of CD4^+^ T cells peaked at 2 weeks post-I/R injury (5, 18). In the present study, we showed that baicalein significantly suppressed Th17 (CD4^+^ IL-17^+^) cell infiltration into the retinas when examined at 2 weeks post-I/R injury compared with vehicle-treated retinas (**Figures 5A, B** **)**. Moreover, since priming of T-cell responses usually occurs in the secondary lymphoid tissues, such as the draining LNs (10), we investigated the T-cell responses in the eye’s draining LNs and defined the proportions of CD4^+^ T-cell subsets in the superior cervical LNs after I/R injury. Compared with the vehicle-treated retinas with I/R injury, baicalein effectively suppressed the activation of Th17 (IL-17^+^) and Th1 (IFN-γ^+^) cells in the superior cervical LNs at 2 weeks post-I/R injury (**Figures 5C-F**). The results indicate that baicalein suppresses CD4^+^ T-cell infiltration into the retina, particularly Th17 cells, as well as their activation in the superior cervical LN after retinal I/R damage.

### Therapeutic Administration of Baicalein Rescues RGCs and Visual Functions in the Retinal I/R Mice Model

**Figure 6:**
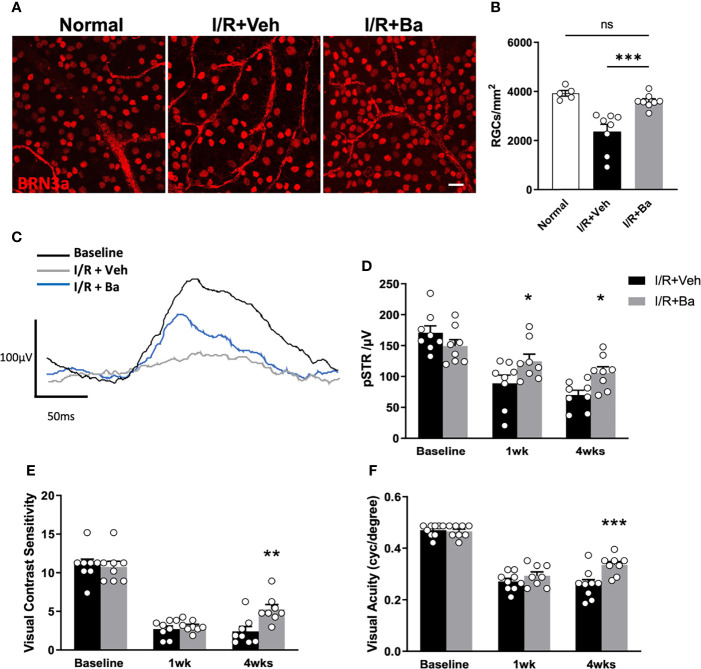
Therapeutic administration of baicalein rescues retinal ganglion cells (RGCs) and visual functions in retinal I/R mice model. **(A)** Representative immunofluorescence photomicrographs of BRN-3a-labeled RGCs (red) and **(B)** quantification of surviving RGCs in whole-mounted retina from normal mice and vehicle or 100 μM baicalein (Ba)-treated mice at 4 weeks post-I/R injury. Scale bar = 20 μm. Representative waveforms **(C)** and quantification **(D)** of pSTR in vehicle or 100 μM baicalein-treated mice at 0, 1, and 4 weeks post-I/R injury. Line color: black = baseline, blue = I/R + 100 μM Ba, and gray = I/R + Veh. Spatial vision of **(E)** visual contrast sensitivity and **(F)** visual acuity from vehicle or 100 μM baicalein-treated mice at 1 and 4 weeks post-I/R injury were shown. *N* = 8–9/group. Data were presented as mean  ± SEM. Each dot represented individual replicates. ns, non-significant difference; *P < 0.05, **P < 0.01, ***P < 0.001, two-way ANOVA with Šídák’s multiple comparisons test.

Lastly, we examined if baicalein presents a therapeutic effect on protecting retinal neurons and visual functions post-I/R injury. Surviving neurons were quantified by immunostaining of retinal flat mounts for a specific RGC marker, BRN3a, at 4 weeks post-I/R (**Figure 6A**). The RGC density in the I/R-injured mice was reduced to 2,369 ± 383 cells/mm^2^ (39.3% loss), compared with that of the normal retina (3,901 ± 145 cells/mm^2^) (**Figures 6A, B** **)**. Weekly administration of baicalein significantly rescued RGCs from I/R-induced injury, with 3,600 ± 137 cells/mm^2^ (7.7% loss) RGC density (**Figures 6A, B** **)**.

To investigate the effects of baicalein on RGC physiological function and spatial vision following I/R injury, ERG and optomotor response (OMR) assessments were conducted respectively at 1 and 4 weeks post-I/R injury. pSTR is a widely used indicator of RGC function as previously described (5, 31, 32). In vehicle-treated eyes, significant and progressive reduction of pSTR amplitude was detected (**Figures 6C, D** **)**. In contrast, baicalein-treated eyes showed significant improvement on pSTR at 1 and 4 weeks after I/R injury (**Figures 6C, D** **)**. Compared with the reduced contrast sensitivity and visual acuity in vehicle-treated mice following I/R injury, baicalein-treated mice exhibited significant improvements in both contrast sensitivity and visual acuity 4 weeks after I/R insults (**Figures 6E, F** **)**. Taken together, these results indicated that weekly intravitreal injections of baicalein can effectively improve RGC survival and enhance RGCs’ physiological function as well as spatial vision.

## Discussion

The present study demonstrated that baicalein exhibits robust anti-inflammatory effects in both human and mouse microglial cells, which were likely achieved by attenuating the phosphorylation of PI3K and NFκB. Proteomic studies further revealed that the anti-inflammatory effects of baicalein involve the inhibition of the IL-1β and IL-17 axis. Administration of baicalein *in vivo* to I/R injured mice resulted in the alleviation of retinal neuroinflammation, as characterized by the suppression of microglial activation, downregulation of IL-1β, subsequent inhibition of Th17 cell infiltration, and activation of Th17 and Th1 cells in LNs. Furthermore, baicalein exhibited therapeutic potential by protecting the physiological functions of RGCs and spatial vision, as well as rescuing over 90% of RGCs following retinal I/R injury. Overall, our results suggested that baicalein acts as a negative regulator of activated immune responses, potentially alleviating microglial inflammatory response-dependent neurodegeneration in retinal I/R injury.

Recent studies have shown that baicalein protects neurons in rats with experimental models of Parkinson’s disease and ischemic stroke injury (33, 34). However, the underlying mechanisms remain unclear. As indicated by previous studies, neuroinflammation triggered by excessive and chronic microglia activation is an important driver to neurotoxicity and degeneration (35, 36). Consistent with previous studies, we found that LPS+ATP or retinal I/R-induced microglial activation increased the expression of pro-inflammatory cytokines. Baicalein effectively suppressed the expression of most cytokines at both the protein and mRNA levels; these included IL-6, IL-1β, and TNF-α, when examined in LPS-primed BV2 cells. In contrast, the vehicle-treated group showed a similar expression profile of cytokine inductions as LPS-stimulated control. While *Gapdh* was selected as a reference and housekeeping gene, some studies reported that its expression was altered in stimulated microglia (37), potentially leading to time-dependent changes (38), whereas other studies showed stable *Gapdh* expression under different experimental conditions (39, 40). Thus, caution must be taken when analyzing the longitudinal changes of cytokine gene levels.

Activation of PI3K/NFκB axis in microglia was reported as one indicator of microglial activation and neuroinflammatory disease (27, 41, 42). Through attenuating PI3K and NFκB signaling, baicalein resolved LPS-induced microglia activation *in vitro* and I/R injury-induced retinal microglia activation *in vivo*, thereby preventing retinal neuron and function loss. The neuroprotective effect of baicalein achieved by inhibiting the NFκB pathway was previously also reported in rats with ischemia stroke injury (43). Proteomic studies were conducted to understand the regulatory mechanisms of baicalein against LPS-induced microglial activation. Previous reports showed that baicalein protects neurons against oxidative stress and lipid metabolism disorders (14, 44). Consistently, our enriched GO and MPO analysis also demonstrated that baicalein mediates the biological processes of oxidation–reduction and lipid metabolic process (**Figures S1A, C** **)**. Additionally, we noted that baicalein also regulates immune system processes and inflammatory responses (**Figures S1A, C** **)**. Specifically, IL-17 signaling, the only pathway suppressed by baicalein (with a *z*-score = 2), is an important pathological trigger in many chronic inflammatory neurological diseases, including ischemic brain injury and Alzheimer’s disease (45, 46). To the best of our knowledge, this is the first report showing an anti-IL-17 signaling pathway by baicalein in retinal I/R injury. Upregulation of IL-17 was detected in retinal ischemia or diabetic retinopathy (47, 48). Targeting IL-17 signaling may present an appealing strategy for treating neuroinflammatory and neurodegenerative diseases (48, 49). Th17 cells are a primary source of IL-17, which infiltrated the retina post-I/R injury; this was particularly alleviated by baicalein treatment as observed using flow cytometry, consistent with the IPA canonical pathway prediction that baicalein suppresses IL-17 signaling. Future phosphoproteomic studies with peptide enrichment strategy are warranted to unravel the involvement of phosphorylated pathways, such as the PI3K/NFκB axis, underlying the baicalein-mediated effects.

We have recently shown that excessive microglia activation-induced retinal neuroinflammation presents as a primary pathological factor leading to retinal degeneration in retinal I/R injury (18). Maintaining the homeostasis of the retinal immune microenvironment is an appealing therapeutic option to alleviate neuronal degeneration and to maintain retinal function, such as through targeting colony stimulator factor 1 receptor (CSF1R) or neutralizing infiltrated CD4^+^ T cells (5, 18, 50). Consistent with other reports (51–55), the upregulated expression of *Iba-1*, *Ptgs2*, *Nox2*, *Tlr4*, and *Ym1*, which are known to be sensitive markers of microglia activation, was detected in injured retina in our study. Remarkably, baicalein significantly suppressed microglial-dependent inflammatory alterations in the retina, including the inhibition of microglia activation markers and pro-inflammatory cytokine expression post-I/R injury. Among those activated microglia markers, the suppression of *Ptgs2* expression by baicalein in I/R injured retina are in agreement with the downregulation of PTGS2 protein observed in proteomics analyses (**Figure 3C**). In addition, the suppression of *Tlr4* and *Il-1β* expression in baicalein-treated I/R injured mouse retina supported the inhibition of upstream regulators of IL-1β and TLR4, as predicted by IPA in our proteomics results (**Figures 3D, E** **)**. To our surprise, *Ym-1*, a marker for anti-inflammatory microglia/macrophage phenotype involved in repair and regeneration (56–58), was downregulated by baicalein treatment in I/R injured retina at 1-week time point, compared with vehicle-treated control. Additional studies are required to determine the precise involvement of YM-1 in mediating baicalein’s protective effects following I/R injury.

Consistent with previous studies, deficits of retinal physiological responses and spatial visual functions commonly occur in retinal I/R injury (5, 18, 59, 60). Our results demonstrated the potent therapeutic capacities of baicalein following I/R insults, as reflected by increased RGC density, retinal physiological functions, visual acuity, and contrast sensitivity. Our findings are in parallel with a previous report in which baicalein pretreatment significantly improved retinal neuron survival in a mouse model of I/R injuries (61). Our studies extended this finding not only by employing the therapeutic administration of baicalein but also demonstrating the improvement of visual behavior performance (visual acuity and contrast sensitivity) after a prolonged period of 4 weeks post-I/R injury. In future studies, the therapeutic effects of baicalein against the subsequent damages triggered by I/R injury should be evaluated.

The present study provides strong evidence supporting the therapeutic potential of baicalein, a natural bioactive flavonoid, from its role as an immune regulator in retinal ischemia. Administration of baicalein not only protects against neuronal loss but also ameliorates spatial vision loss. Given that baicalein has outstanding anti-inflammatory and neuroprotective capacities, it could serve as a potential treatment for various neuroinflammation-dependent neurodegenerative diseases including retinal I/R injury.

## Data Availability Statement

The mass spectrometry proteomics data have been deposited to the ProteomeXchange Consortium via the PRIDE partner repository with the dataset identifier PXD030447.

## Ethics Statement

All animal procedures were reviewed and approved by the Schepens Eye Research Institute Animal Care and Use Committee and conformed to the standards of the National Institute of Health and the Association for Research in Vision and Ophthalmology.

## Author Contributions

DC, K-SC, and C-WD conceived, conceptualized, designed, and supervised the general project. DC, K-SC, C-WD, and LP wrote and edited the manuscript. LP, YS, MY, and JT performed the experiments and collected the data. LP, YS, and CL performed mass spectrometry and proteomics data analysis. LP, JT, SZ, and IY performed all the animal experiments, masked the data analysis, and edited the manuscript. C-HT edited and commented on the manuscript.

## Funding

The research reported in this publication was supported by Mass Eye and Ear Summit Fund, Harvard NeuroDiscovery Center Grant, NEI R01 EY025259, R01 EY031696, and R44 EY025913 (DC); BrightFocus Foundation and The Glaucoma Foundation (K-SC); Health Medical Research Fund (16172571) (C-WD); PolyU internal grants (UAGF, UAHG) (C-WD); Dr. George Cheng’s generous donation (C-WD); Shenzhen Science and Technology Innovation Commission, JCYJ20180507183409601 (CL); Henry G Leong Professorship in Elderly Vision Health (C-HT); PolyU Postgraduate Studentship (LP; YS); and NIH/NEI core Grant for vision Research P30EY03790 (Schepens Eye Research Institute).

## Conflict of Interest

DC is employed as a consultant by Boston Pharma (Cambridge, MA), FireCyte Therapeutics (Delaware), iLumen Scientific (Delaware), and PriMed (Sichuan, China). K-SC is employed as a consultant by Sunregen (Switzerland). A provisional patent application related to this work was filed by C-WD, DC, K-SC, C-HT, and LP.

The remaining authors declare that the research was conducted in the absence of any commercial or financial relationships that could be construed as a potential conflict of interest.

## Publisher’s Note

All claims expressed in this article are solely those of the authors and do not necessarily represent those of their affiliated organizations, or those of the publisher, the editors and the reviewers. Any product that may be evaluated in this article, or claim that may be made by its manufacturer, is not guaranteed or endorsed by the publisher.

## Supplementary Material

Supplementary Figure 1 Bar charts of top 10 most significant GO terms in category of biological process **(A)** and enriched MPO terms **(B)** ranked by Log_10_P-value and projected based on 742 DEPs between LPS + Veh and Ctrl. Bar charts of the top 10 most significant GO terms in category of biological process **(C)** and enriched MPO terms **(D)** ranked by Log_10_P-value and projected based on 28 DEPs between LPS + Ba vs LPS + Veh. All DEPs were with cutoff at P-value<0.05 and FC > ± 1.2.

Supplementary Figure 2 Representative immunofluorescence images of IBA-1 labeled microglial cells (green) on flat mount retina from vehicle (Veh) or 100μM baicalein (Ba) treated mice at 4 week post-I/R. Scale bar = 20 μm.

## Abbreviations

IL-6, interleukin 6; IL-1β, interleukin 1 beta; TNF-α, tumor necrosis factor α; IL-17, interleukin 17; I/R, ischemia/reperfusion; NFκB, nuclear factor-κB; PI3K, phosphoinositide 3-kinase; RGC, retinal ganglion cell; CNS, central nervous system; SB, *Scutellaria baicalensis* Georgi; Ba, baicalein; LPS, lipopolysaccharides; ATP, adenosine triphosphate; FDR, false discovery rate; MPO, Mammalian Phenotype Ontology; GO, Gene Ontology; ERG, electroretinography; pSTR, positive scotopic threshold responses; OMR, optomotor response; LN, lymph node.
