# Supplementary figures and images for: Baicalein—A Potent Pro-Homeostatic Regulator of Microglia in Retinal Ischemic Injury
Source: Front Immunol. 2022 Feb 21;13:837497. doi: 10.3389/fimmu.2022.837497 (PMC8899187; doi:10.3389/fimmu.2022.837497)

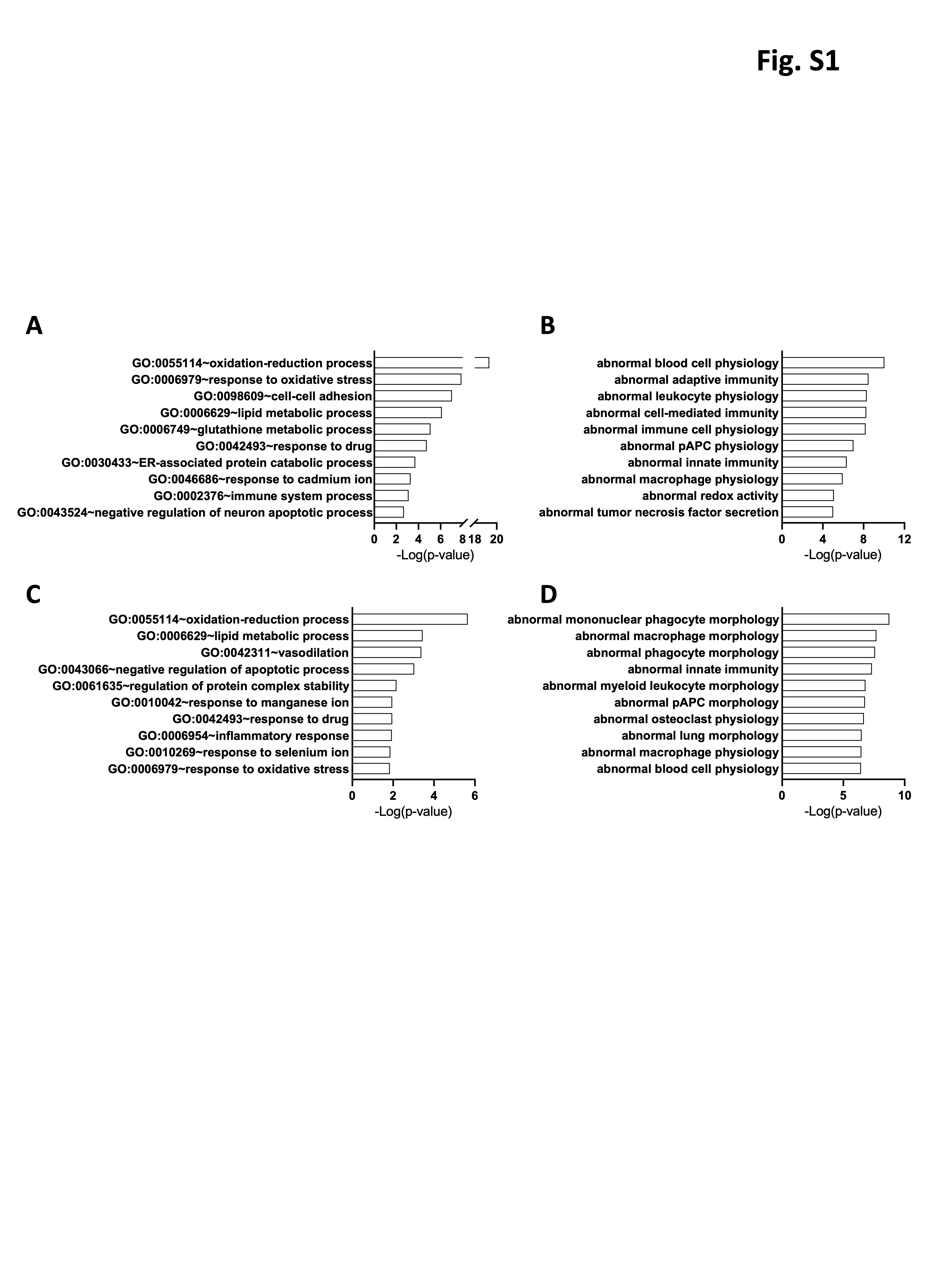

Supplement: Supplementary Figure 1 — Bar charts of top 10 most significant GO terms in category of biological process (A) and enriched MPO terms (B) ranked by Log10P-value and projected based on 742 DEPs between LPS + Veh and Ctrl. Bar charts of the top 10 most significant GO terms in category of biological process (C) and enriched MPO terms (D) ranked by Log10P-value and projected based on 28 DEPs between LPS + Ba vs LPS + Veh. All DEPs were with cutoff at P-value<0.05 and FC > ± 1.2. [file Image_1.jpeg]

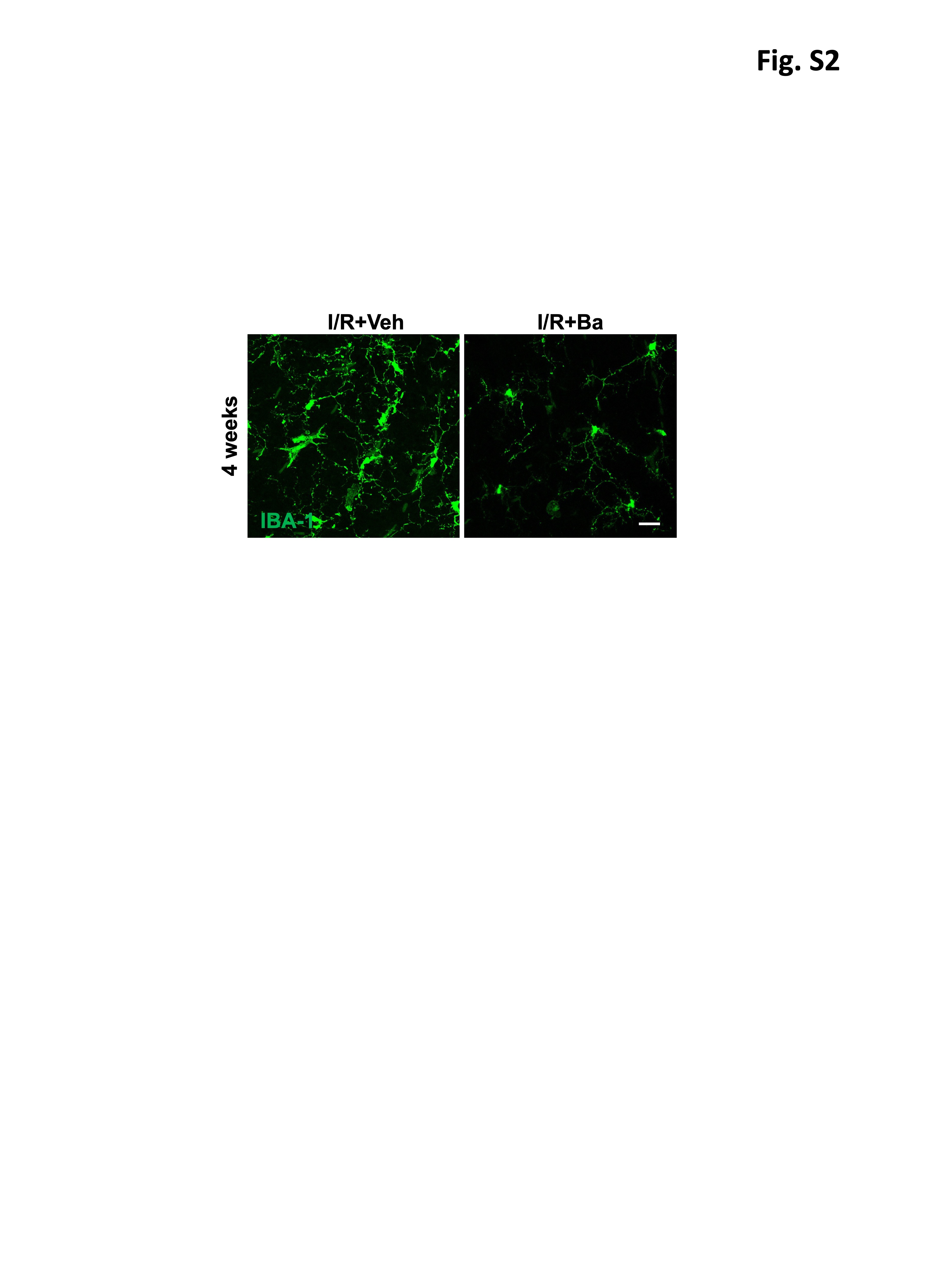

Supplement: Supplementary Figure 2 — Representative immunofluorescence images of IBA-1 labeled microglial cells (green) on flat mount retina from vehicle (Veh) or 100μM baicalein (Ba) treated mice at 4 week post-I/R. Scale bar = 20 μm. [file Image_2.jpeg]
